# Normal Saline Versus Low Chloride Solutions in Treatment of Diabetic Ketoacidosis: A Systematic Review of Clinical Trials

**DOI:** 10.7759/cureus.21324

**Published:** 2022-01-17

**Authors:** Ahmad Jahangir, Abdullah Jahangir, Fasih Sami Siddiqui, Muhammad Rafay Khan Niazi, Fahad Yousaf, Marwah Muhammad, Syeda Sahra, Aneeqa Javed, Muhammad Ans Sharif, Qasim Zafar Iqbal, Michael Krzyzak

**Affiliations:** 1 Medicine, Mayo Hospital, Lahore, PAK; 2 Medicine, Staten Island University Hospital, New York City, USA; 3 Medicine, Northwell Health, New York City, USA; 4 Internal Medicine, Jinnah Hospital, Lahore, Lahore, PAK; 5 Internal Medicine, Northwell Health, New York City, USA; 6 Endocrinology and Diabetes, Ochsner Medical Center, New Orleans, USA

**Keywords:** insulin, normal saline, lactated ringer, hyperglycemia, diabetes mellitus, diabetic ketoacidosis, dka, crystalloid solutions

## Abstract

Traditionally, normal saline solution (NSS) has been the fluid of choice in diabetic ketoacidosis (DKA) patients, but the NSS is an acidic fluid and may lead to the delayed resolution of DKA. A systemic review search was conducted on PubMed, Embase, and Central Cochrane Registry to compare the efficacy of low chloride solutions with normal saline solution in DKA resolution. Randomized clinical trials with normal saline as a control arm and low chloride solutions as an intervention arm were included. Four studies were included in the analysis. The investigated outcomes, including time to resolution for DKA and duration of insulin infusion, varied depending on the endpoint were reported in the studies. Overall, balanced solutions were generally associated with faster correction of pH. The time to reach overall DKA endpoints was comparable in both groups. We concluded that crystalloid solutions may be used as an initial resuscitation fluid in the DKA population and may lead to earlier resolution of acidosis. More clinical trial data is required to reach statistical significance for the hypothesis.

## Introduction and background

Diabetic ketoacidosis (DKA) is an acute emergency consisting of a triad of hyperglycemia, ketonemia, and high anion gap metabolic acidosis (HAGMA) [1]. The pathogenesis involves hyperglucagonemia and insulinopenia, leading to the increased production of ketone bodies, which causes the development of HAGMA when the body's buffering capacity is overwhelmed [2]. Current data suggest a prevalence of DKA ranging from approximately 50 to 100 events per 1000 adult patients with Type 1 diabetes mellitus (DM), with a higher prevalence in women, non-whites, and the patient being treated with insulin injections (as opposed to infusion pumps) [3]. From 2003 to 2014, the total number of hospital discharges with the primary diagnosis of DKA increased from 118,808 to 188,965, and the resulting national bill in the United States for DKA rose from $2.2 billion (inflation-adjusted) in 2003 to $5.1 billion in 2014 [4].

DKA is a volume-depleted state, and a typical adult patient will have a water deficit of 100 mL/kg [5]. The goals of DKA management include volume expansion via intravenous (IV) fluids along with correction of ketosis and electrolyte repletion via insulin drip and parenteral electrolyte supplementation. Normal saline solution (NSS) is one of the most frequently used IV crystalloid solutions globally, with more than 200 million liters used per year in the US alone [6]. However, using NSS may lead to hyperchloremia and acidosis, which can potentially disrupt coagulation, myocardial contractility, immune function, and renal function with renal arteriolar vasoconstriction precipitating oliguria and deceleration of acidosis correction [7]. There has been debate about the best choice of fluid for DKA resuscitation, and in this analysis, we review different clinical trials and publications where balanced/low chloride IV solution (such as lactated Ringers (LR), plasmalyte) is compared with high chloride IV solution (normal saline solution).

## Review

Methods

Literature Search

A broad literature search was done on PubMed, Cochrane and Embase using the terms "DKA," "Normal Saline," "Lactated Ringer," and "Crystalloid Solutions." The search was not limited to any region or any language if the English translation were available. The cut-off date for this search was Jan 1, 2021. Relevant articles from recent conferences were also included. No age filter was applied for the patient population.

Eligibility Criteria

Only those studies were included in the review which: 1. Involved Normal Saline as the control arm; 2. Had low chloride solutions as the intervention arm; 3. Are original clinical studies with the human population; 4. Have reported the safety and efficacy outcomes.

Study Selection

Two independent reviewers reviewed relevant studies (AJ, SS) based on the title and abstract. Then the potential articles obtained from the first review were screened again with full text by the same reviewers. The risk of bias for selected papers was assessed using Cochrane collaborative tool and classified into high, uncertain, and low.

Data Extraction and Analysis

Data extraction was done using pre-specified tables of baseline characteristics, time to resolution of DKA, duration of insulin infusion. The parameters recorded were time to DKA resolution and the duration of insulin infusion required. For calculation of mean difference, Comprehensive Meta-analysis software Version 3 was used. In papers reporting the continuous variables as medians, it was assumed to be equivalent to the mean, and SD estimation was obtained by dividing the interquartile difference by 1.35. The data is demonstrated in Table 1, Table 2, and Table 3.

**Table 1:** Primary characteristics and outcomes of studies included DKA: diabetic ketoacidosis; ESRD: end-stage renal disease; CKD: chronic kidney disease; GCS: Glasgow coma scale; AKI: acute kidney injury; ED: emergency department; RCT: randomized controlled trial; ISPAD: International Society of Pediatric and Adolescent Diabetes; HCO3: bicarbonate; Cl: chloride; Na: sodium; K: potassium; SC: subcutaneous; PO: per os; PICU: pediatric intensive care unit; AG: anion gap

Study	Study type	Participants	Patient Population	Inclusion	Exclusion	Intervention (Included in Analysis)	Primary Outcome	Secondary Outcomes
Self et al. 2020 [8]	subgroup analysis of 2 companion trials SALT-ED and SMART	172	Adults	Age>18 Clinical Diagnosis of DKA Lab Values consistent with DKA present in ED	Transfer from another hospital Admission to cardiac or Neurologic ICU	Balanced Crystalloid (n=94) vs Normal Saline (n=78)	Time between ED presentation and DKA resolution	Time between Initiation and discontinuation of insulin infusion
Van Zyl et al. 2011 [9]	Parallel Double-Blind RCT	54	Adults	Age >18 years Lab Diagnosis of DKA	ESRD or Lactic Acidosis Ionotropic or Ventilatory support More than 1L Fluids given prior to intervention	LR (n=27) vs Normal Saline (n=27)	Time to Resolution of DKA	x
Williams et al. 2020 [10]	Parallel Double Blind RCT	66	Pediatrics	Age 1 month -12 years DKA as per ISPAD 2014	CKD, Liver Disease Cerebral edema (GCS < 8) Pre intervention fluids or insulin	Plasmalyte (n=34) vs Normal Saline (n=32)	New-Onset AKI	Time for resolution of DKA Change in Cl, HCO3, pH All-cause mortality
Yung et al. 2017 [11]	Parallel Double Blind RCT	77	Pediatrics	Moderate to Severe DKA	GCS<11 Mechanical Ventilation Corrected Na<130 K>5.5	Hartman Solution (n=38) vs Normal Saline (n=39)	Time to reach Bicarb>15	Time to reach Venous pH>7.3 Time to SC insulin Time to PO intake Time to change in fluid Total insulin requirement/kg Length in PICU Time to normalization of AG

**Table 2:** Duration to reach outcomes in the adult population DKA: diabetic ketoacidosis; AG: anion gap; LR: lactate Ringer's; ADA: American Diabetes Association

Study	Intervention		Time to DKA resolution (hours)		
		End Point	Normal Saline	Balanced Fluids	Mean difference
Self et al. 2020 [8]	Balanced Crystalloid (n=94) vs Normal Saline (n=78)	Glucose < 200mg/dL and 2 of the following: Bicarb <= 15 mEq/L, venous pH > 7.3, and AG <= 12 mEq/L or patient discharged from hospital	16.9(11.9-34.5)	13 (9.5-18.8)	-3.9 (-7.611 to -0.189)
Van Zyl et al. 2011 [9]	LR (n=27) vs Normal Saline (n=27)	pH >7.32	11.38 (7.25-18.25)	9 (5-15.67)	-2.38 (-5.670 to 0.910)
		Glucose 14 mmol/L	5 (3.92-7)	6.83 (4-9)	1.83 (0.191 to 3.469)
		Combined End Point (ADA 2006)	14.08 (9.38-23)	14.5 (7.02-27.5)	0.420 (-6.450 to 7.290)

**Table 3:** Duration to reach outcomes in the pediatric population DKA: diabetic ketoacidosis

Study	Intervention		Time to DKA resolution (hours)		
		End Point	Normal Saline	Balanced Fluids	Mean difference
Williams et al. 2020 [10]	Plasmalyte (n=34) vs Normal Saline (n=32)	pH > 7.3, bicarbonate> 15 mEq/L & normal sensorium	16(8-20)	14.5(12-20)	-1.5 (-5.121 to 2.121)
Yung et al. 2017 [11]	Hartman Solution (n=38) vs Normal Saline (n=39)	Bicarbonate>15	8.6(2.3)	6.2(4.7)	-2.4 (-4.046 to -0.754)
		pH >7.3	8.5(2.8)	7.5(1.8)	-1 (-2.054 to 0.054)
		Normal Anion Gap	6.3(1.7)	6.7(1.6)	0.4 (-0.338 to 1.138)

Bias

**Figure 1:**
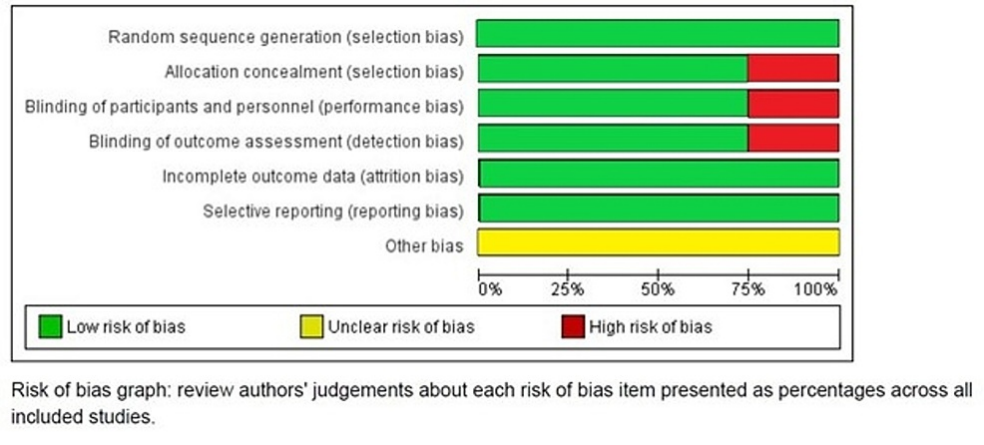
Risk of bias graph in studies included

**Figure 2:**
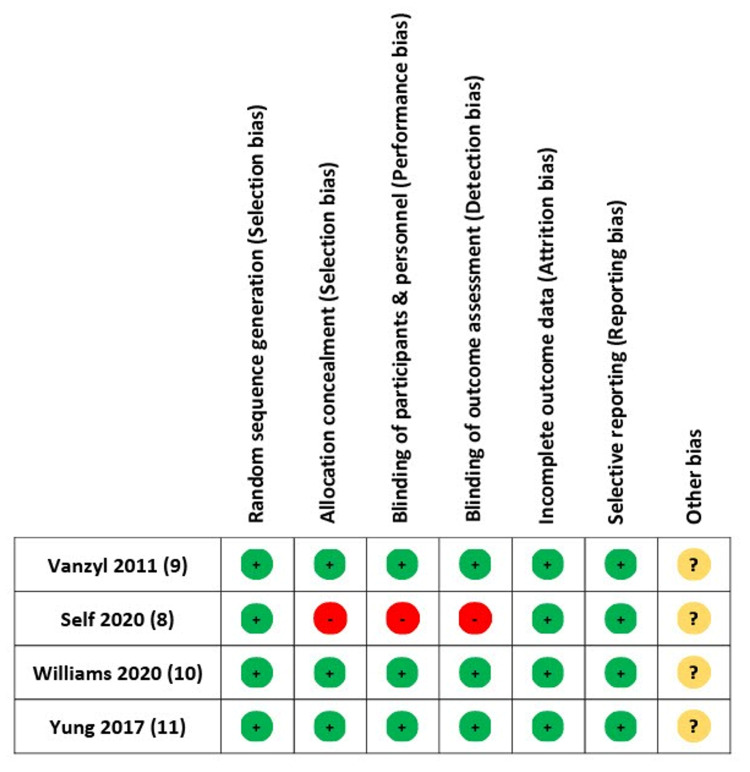
Risk of bias summary for the included studies

The risk of bias in studies included was also calculated (Figures 1, 2).

Results

The literature search yielded a total of 227 articles. After excluding 12 duplicates, 215 articles were screened based on titles and abstracts. After the first screening, 20 studies were found potentially useful for our review and full texts of these articles were reviewed, and further 16 articles were excluded due to one of the following reasons: review papers (n=6), articles without relevant outcomes (n=3), abstracts (n=4), case reports (n=2) and suspended trials (n=1). This study selection and screening process are depicted in the PRISMA (Preferred Reporting Items for Systematic Reviews and Meta-Analyses) flowsheet. After screening, four clinical trials were included in the final review.

**Figure 3:**
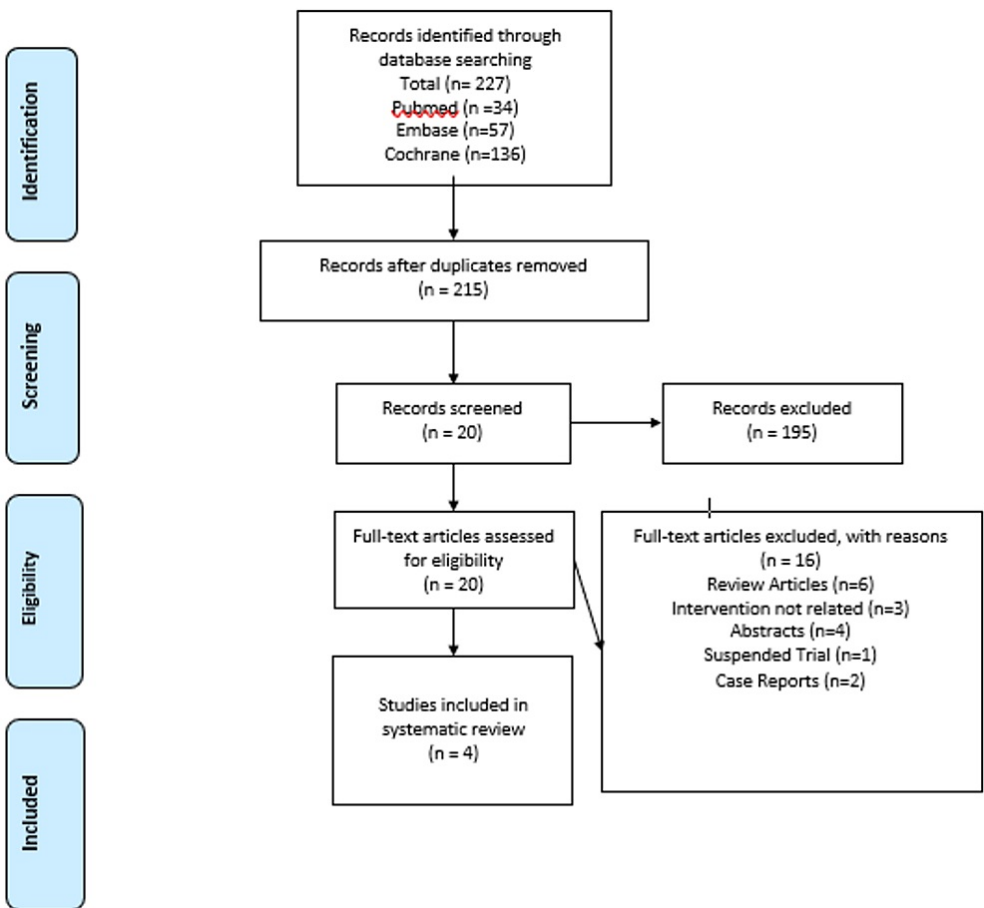
PRISMA 2009 flow sheet for selection of studies PRISMA: Preferred Reporting Items for Systematic Reviews and Meta-Analyses

Time to DKA Resolution

Adult Population: Two studies reported the duration to reach different endpoints in the DKA population, as listed in Table 2.

Pediatric Population: Two studies in pediatric populations reported time to different endpoints for DKA resolution listed in Table 3.

Duration of Insulin Infusion

One study reported the duration of insulin infusion in adults (Self et al. [8]) for DKA. The study reported normal saline requiring more time, 13.4 h (11-17.9), than 9.8 hours (5.1-17) in balanced solutions before switching to subcutaneous insulin. The difference in means between the groups was -3.6h (-5.811 to -1.389), p=0.001. Similarly, one study reported the duration of insulin infusion in the pediatric population (Yung et al. [11]). The study reported a shorter duration required for insulin infusion in the balanced crystalloid group 6.2h (SD 4.7) compared to normal saline 8.6h (SD 2.3). The mean difference between the groups was -2.4h (-4.046 to -0.754), p=0.004.

Amount of Volume Infused

Three studies in total reported the amount of volume infused of the respective solutions. No statistically significant difference was found between the intervention and control groups with regards to infused volume (Table 4).

**Table 4:** Volume infused of respective solutions

Study	Volume Infused (ml)			
	Control	Interquartile Range	Intervention	Interquartile range
Self et al. 2020 [8]	4927	(3324-6026)	4267	(3000-7090)
Williams et al. 2020 [10]	1190	(810-1857)	1200	(760-1785)
Yung et al. 2017 [11]	2167	(1114-2997)	1771	(988-2623)

Mortality

Three studies reported mortality in their respective treatment and control populations. Self et al. reported no death in control, while Williams et al. reported two deaths in the intervention arm [8]. (Table 5)

**Table 5:** Reported mortality

Study	Reported Mortality	
	Control	Intervention
Self et al. (2020 )[8]	1	0
Vanzyl et al. (2012) [9]	0	0
Williams et al. (2020) [10]	0	2

Hyperkalemia

Self et al. (2020) reported an incidence of new-onset hyperkalemia with 18 episodes in control vs. 11 in the intervention arm [8].

Hypokalemia

In one study, Self et al. (2020) reported an incidence of new-onset hypokalemia with 15 episodes in control vs. 9 in the intervention arm [8].

Change in Chloride Levels

In one study, Van Zyl et al. (2012) reported a change in chloride at the end of DKA resolution with a mean rise of chloride by 7.72 in control vs. 7.18 in the intervention arm [9].

Discussion

Per American Diabetes Association (ADA) guidelines, extracellular volume expansion via IV fluid infusion is the first step in DKA treatment. Fluid replacement stabilizes cardiovascular status while also enhancing insulin sensitivity by diminishing plasma osmolality (Posm), reducing vasoconstriction and improving perfusion, and lowering counter-regulatory hormones [12].

NSS has usually been the fluid of choice for diabetic ketoacidosis, and its use is advocated in clinical guidelines [5, 12, 13]. However, despite its frequent usage, NSS has potential shortcomings, including a pH of 5.5 and high chloride content that cause hyperchloremic metabolic acidosis with large-volume administration. The content of chloride in normal saline is 154. As plasma chloride concentration increases, bicarbonate, and the other primary anions in plasma decrease due to dilution and maintenance of electro-neutrality, resulting in hyperchloremic metabolic acidosis [6, 14-16]. Hence, using NSS for DKA could worsen metabolic acidosis, thereby prolonging recovery time and incidence of acute kidney injury.

In contrast to NSS, a balanced salt solution is akin to serum in chloride concentration and pH and does not induce metabolic acidosis [6, 17-19]. It is hypothesized that such solutions may expedite the resolution of DKA compared to NSS, thereby improving patient recovery and reducing healthcare costs.

Our study reveals that low chloride solution may be a better alternative to NSS by offering reduced recovery time in DKA patients. Findings from Van Zyl et al. failed to show any statistically significant superiority of LR in DKA resolution (based on ADA criteria) and normalization of pH [9]. However, the LR group took a significantly longer time to lower blood glucose below 14mmol/L (152 mg/dL), at which point the treating clinician was allowed to continue with any dextrose or glucose-containing fluid according to personal inclination. One explanation for this delay in glucose control can be attributed to lactate from lactated ringer solution being used as a substrate for ongoing gluconeogenesis [11]. Contrary to Van Zyl et al., Self et al. statistically significant shorter time to DKA resolution (based on ADA criteria or discharge from the hospital) and a shorter insulin infusion requirement in the balanced crystalloids group instead of the NSS group. Analysis of results reveals balanced crystalloids were associated with an absolute reduction of roughly 4 hrs and a relative reduction of roughly 20%-30% in the time to DKA resolution and discontinuation of insulin infusion [8].

In their study in the pediatric population, Williams et al. (2020) reported new or progressive acute kidney injury (AKI) incidence as the primary outcome. Even though the primary outcomes remained statistically insignificant, mild chloride elevation in both groups was not associated with increased AKI [10]. No statistical difference was noted in time to achieve a composite DKA endpoint in both groups. Yung et al. (2017) also studied pediatric DKA patients and reported the primary outcome as the time to reach venous bicarbonate of 15 mmol/L. On analysis of results, the study did show association of plasmalyte with significantly lower time in achieving bicarbonate levels of >15. However, it failed to show any significant time difference in normalization of pH despite strong deviation towards balanced solutions or closure of anion gap [11].

Overall, it can be safely said that the low chloride solutions were comparable in outcomes to normal saline solutions. Results showed a deviation toward earlier pH correction in both adults and pediatric populations, but overall time to combined endpoints remained similar. The findings are limited statistically due to a small number of studies and a small patient population. There is growing evidence that balanced solutions are associated with favorable outcomes in various patient populations [20-23]. Even limited benefits, which were highlighted by our study when applied to a large global DKA population, can benefit the overall healthcare system.

Based on this review paper, we have ascertained that low chloride solutions, if not superior, are an acceptable alternative to normal saline as current data does not show any harm of using low chloride solution when managing DKA. More clinical trials are needed using low chloride solutions as a choice of fluid to see if they can improve recovery time for DKA patients.

## Conclusions

Low chloride solutions are an acceptable alternative to normal saline in the management of DKA. Their use may be associated with faster normalization of pH but delayed correction of hyperglycemia.
